# Up-Regulation of miRNA-146a in Progressive, Age-Related Inflammatory Neurodegenerative Disorders of the Human CNS

**DOI:** 10.3389/fneur.2014.00181

**Published:** 2014-09-29

**Authors:** Peter N. Alexandrov, Prerna Dua, Walter J. Lukiw

**Affiliations:** ^1^Russian Academy of Medical Sciences, Moscow, Russia; ^2^Department of Health Information Management, Louisiana State University, Ruston, LA, USA; ^3^Department of Neurology, Louisiana State University Health Science Center, New Orleans, LA, USA; ^4^LSU Neuroscience Center and Department of Ophthalmology, Louisiana State University Health Science Center, New Orleans, LA, USA

**Keywords:** prion disease, Alzheimer’s disease, age-related macular degeneration, miRNA-146a, neuroinflammation, innate-immune response

## Overview

The human brain- and retinal-resident microRNA-146a (miRNA-146a) is an inducible, NF-kB-regulated small non-coding RNA (sncRNA) whose increased expression is associated with pro-inflammatory neurodegeneration in Alzheimer’s disease (AD), age-related macular degeneration (AMD), and prion disease (PrD). In AD, AMD, and PrD miRNA-146a modulates the innate-immune response, inflammation, and the microglial activation state. This short paper will review and comment on the role of miRNA-146a signaling and how it underlies common molecular-pathogenetic mechanisms in each of these progressive, age-related neurological disorders for which there are currently no effective treatment or cure.

## microRNA-146a

The 22 nucleotide miRNA-146a (miR-146a; hsa-miR-146a-5p; 5′-UGAGAACUGAAUUCCAUGGGUU-3′; 59% A + U; NR_029701; http://atlasgeneticsoncology.org/Genes/GC_MIR146B.html), is one of the most intensively studied small non-coding RNAs (sncRNAs) in all of human neurobiology. Encoded from a single locus at chromosome 5q33.3 in humans and at chromosome 11q in mice, miRNA-146a is a rapidly induced, pro-inflammatory miRNA with a relatively short half-life of about 1.5–2 h in human brain cells and tissues (1–4). This unique member of the miRNA-146 gene family was initially described as being significantly up-regulated after microbial endotoxin, lipopolysaccharide (LPS), or cytokine stimulation of THP1 cells (monocytes; originally derived from an acute monocytic leukemia patient) and under transcriptional control by NF-κB; shortly thereafter this inducible miRNA-146a was found to be up-regulated by metal sulfate-generated reactive oxygen species (ROS), by pro-inflammatory cytokines (such as IL-1β and TNFα) and amyloid peptides (such as Aβ42 peptides) in human primary neuronal-glial (HNG) co-cultures and microglial (HMG) cells (4–7). Each of these independent studies showed the targeting of miRNA-146a to the mRNAs encoding signaling proteins involved in the innate immune and inflammatory response, including complement factor H (CFH) and the interleukin-1 receptor-associated kinase 1 (IRAK-1; the gene partially responsible for IL-1-induced up-regulation of the transcription factor NF-kB; (1–4); see below).

Sequencing and promoter analysis of the human miRNA-146a gene subsequently identified three functional and conserved NF-κB binding sites upstream of the miRNA-146a gene, and combined with functionality and NF-kB-inhibition assays was the first NF-κB-regulated miRNA gene identified in the human brain and central nervous system [CNS; (2–4)]. Interestingly, while miRNA-146a is detectable in mouse and human brain and CNS tissues and primary HNG co-cultures, the most significant miRNA-146a abundances have been found to be in human astroglial (HAG) and microglial (HMG) cells, the later representing the “resident scavenging macrophages” of the brain, and key participants in the brain’s innate-immune and inflammatory response (3–9). While expressed modestly in the brain and retina, miRNA-146a can be induced 2- to 10-fold or more in cultured brain cells where miRNA-146a is basally expressed, after the application of several different classes of physiological stressors including treatments with herpes simplex virus (HSV-1), neurotoxic metal sulfates (such as aluminum sulfate at nanomolar concentrations), microbial endotoxins including LPS, pro-inflammatory cytokines or amyloid beta (Aβ) peptides (1–5, 10).

Interestingly, human miRNA-146a has a related miRNA-146b isotype (miR-146b; hsa-miR-146b-5p; 5′-UGAGAACUGAAUUCCAUAGGCU-3′; 59% A + U; NR_030169) encoded at chromosome 10q24.32 (in mice on chromosome 19q); these two miRNAs have an identical seed (primary recognition) region and differ by only two ribonucleotides in the primary sequence of their stem-loop secondary structures (1–3, 10–12). Notably, it is a change in just two nucleotides in the 3′ end, from miRNA-146b (5′-UAGGCU-3′) to miRNA-146a (5′-UGGGUU-3′), which may confer enhanced specificity of miRNA-146a for mRNA targets involved in the innate-immune response of the CNS, and/or the ability to be induced and/or processed by different pro-inflammatory cytokines [(8, 9, 13); unpublished observations].

## Alzheimer’s Disease

According to the World Health Organization, the total number of people with dementia worldwide is currently estimated to be about 36 million, and this number is expected to almost double by 2030, reaching 66 million, and triple by 2050, reaching 100 million (14). Alzheimer’s disease (AD), the leading cause of this dementia, is a progressive, age-related, and ultimately fatal neurological disorder associated with dysfunctional gene expression in the limbic system and entorhinal cortex of the brain that drives amyloidogenesis, pro-inflammatory signaling, and related AD-type neuropathology (15–18). Of all AD cases approximately 5% may be attributed to familial gene mutations while 95% occur sporadically, i.e., are of idiopathic or unknown origin (7, 14).

Amyloidogenesis involves the progressive generation and aggregation of 42 amino acid amyloid beta (Aβ42) peptides and other amyloidogenic peptides into dense, insoluble senile plaques whose recognition by brain cell microglia instigates a pro-inflammatory microglial response and the release of reactive oxygen species (ROS) and pro-inflammatory cytokines (3, 4, 7, 19). The first evidence of sncRNA involvement in sporadic AD reported mis-regulated levels of a polyadenylated brain cytoplasmic ~200 nucleotide (BC200) sncRNA in cases of AD, non-AD dementia, and controls (20). BC200 was found to be down-regulated and reflective of deficits in the abundance of neuron-specific transcripts, consistent with the idea that sporadic AD was characterized by a deficit in the generation of primary brain gene transcription products (20–25). The next reports of specific miRNA up-regulation in AD brain and blood serum did not appear until about 15 years later wherein a brain abundant miRNA-146a was one of the first miRNAs found (i) to be elevated in anatomical regions of the brain affected by the AD process but not in control regions (such as the thalamus and brain stem) of the same brain, or (ii) to be induced by AD-relevant stressors, such as the pro-inflammatory cytokines interleukin 1-beta (IL-1β) or tissue necrosis factor alpha (TNFα) and Aβ42 peptides, or combinations of these noxious factors which are pathologically abundant in the AD brain (6, 7, 21–25).

To date confirmed targets of miRNA-146a include key AD-relevant members of the innate-immune system including the 155 kDa sialic-acid containing glycoprotein immune repressor complement factor H (CFH), the membrane spanning beta-amyloid precursor protein (βAPP)-associated TSPAN12, and the inflammation mediator interleukin receptor-associated kinase IRAK-1 (1). Interestingly, pathologically up-regulated miRNA-146a, as seen in AD brain or pathologically stressed primary HNG co-cultures results in (i) CFH down-regulation and a stimulation of innate immune and inflammatory pathways (10, 25); (ii) down-regulation of TSPAN12 that drives a propensity for the massive production of Aβ42 peptides from βAPP (26); and (iii) down-regulation of IRAK-1 with a compensatory up-regulation of IRAK-2 (1, 4, 10). It is important to point out (i) that multiple NF-kB-regulated miRNAs such as miRNA-9, miRNA-34a, miRNA-125b, and miRNA-155 may have additional or ancillary roles in the pathological regulation of CFH, TSPAN12, and IRAK-1 in the AD brain and (ii) that miRNA abundance and complexity varies among both human cell types and tissues, and there are also obvious variations in pathogenic miRNA expression among various human populations (26–29).

## Age-Related Macular Degeneration

Age-related macular degeneration (AMD) is an advancing, proliferative degeneration of retinal pigment epithelial, ganglion, and other related cells of the human macula, a highly specialized centralized region of the retina, resulting in the progressive loss of vision near the center of the visual field (8, 30–34). AD and AMD share many common pathological pathways including the appearance of dense, insoluble, Aβ42 peptide-enriched lesions (called drusen in AMD and called senile plaques in AD), a disruption in complement signaling including CFH loss-of-function or down-regulation, and the up-regulation of pro-inflammatory sncRNAs that include, prominently, miRNA-146a (32–35). Importantly, the common neuroectodermal origins of the limbic system, neocortex, and retina may predispose each of these highly integrated, multi-neuronal layered structures to progressive age-related functional impairment, including the involvement of shared pathogenic pathways that drive the development and “spreading” of amyloidogenesis and pro-inflammatory neurodegeneration.

As fore-mentioned, CFH plays an integral role in the regulation of the complement-mediated immune system that is involved in the first line of microbial defense against many pathogens, innate-immune complex processing, and programed cell death. CFH is emerging as an unexpected key player in both AD and AMD (10, 25, 30, 31, 36, 37). Activation of the complement system results in a proteolytic cascade eventually forming the membrane attack complex (MAC) leading to cell membrane perforation, lysis, and the dissolution of cellular contents, and a soluble brain- and retinal-abundant CFH normally protects host cells from unrestrained complement activation (31–34, 36).

Interestingly, the Y402H CFH loss-of-function mutation linked to AMD in many human populations may produce sufficient amounts of a non-functioning CFH, and this may be pathologically equivalent to insufficient amounts of a functional CFH protein, as is observed in sporadic AD, and perhaps other inflammatory degenerative and dementing diseases including Down’s syndrome [Trisomy 21; (10, 25, 37)]. Indeed common CFH deficits in AMD and AD underscore the important role of innate-immune system regulation and complement signaling in these age-related progressive, inflammatory neurodegenerative diseases of the CNS. It is further noteworthy that human prion protein (PrP), an endogenous glycosylphosphatidylinositol (GPI)-anchored or transmembrane protein expressed in neurons strongly interacts with CFH, and the CFH-PrP complex pathologically super-activates complement via the classical and alternative pathways, leading to MAC formation and progressive cell dysfunction and ultimately, cell death (38).

## Prion Disease

Prion diseases encompass a family of self-replicating prion protein (PrP) amyloid and related aggregates – driving pathophysiological conditions that are the primary causative factor of a number of progressive neurological diseases in mammals including humans (39–42). The first report of up-regulated miRNA-146a in prion disease was published in 2008 in a murine model of scrapie, and miRNA-146a was subsequently reported to be significantly up-regulated in the two rare human prion diseases sporadic Creutzfeldt-Jakob disease (sCJD) and Gerstmann–Straussler–Scheinker (GSS) syndrome (40–42). In prion disease miRNA-146a and other NF-kB-up-regulated, inducible miRNAs have been found to target the expression of genes involved in intracellular protein-degradation pathways and signaling pathways related to cell death, synapse function and neurogenesis as well as brain genes modulating microglial function by regulating their activation state during PrP-induced neurodegeneration (39–42). Interestingly, human prion diseases such as sCJD and GSS are highly similar to neurological diseases such as AD involving a significant pro-inflammatory and amyloidogenic component linked to progressive memory, cognitive, and behavioral deficits in the affected patient (39, 41).

## Concluding Remarks

Our perceptions on the relevance and gene expression mechanisms of up-regulated miRNA-146a signaling in progressive, pathologically similar, human neurological diseases continue to evolve. It is now generally accepted that the primary mode of pathological miRNA action in the brain is to recognize and bind to complementary ribonucleotide sequences in the 3′-prime un-translated region (3′-UTR) of their target messenger RNAs and in doing so, down-regulate their expression (1–3, 10–13, 40, 43). Increased expression of miRNA-146a and down-regulation in the expression of miRNA-146a target genes are strongly associated with AD, AMD, and PrD disease phenotype and symptomology, both in cultured cell or animal models for that disease and in the human disease itself (31, 37, 44). miRNA-146a or families of other miRNA-146a-related miRNAs may orchestrate multiple deficits in multiple mRNA targets to coordinately affect the expression of families of brain-relevant innate-immune and inflammatory genes that are related in function in disease initiation, development, and propagation. It is noteworthy that the speciation and complexity of miRNA-146a-related families may differ slightly among different types of CNS cells and tissues, and even among the same cells and tissues of different human populations (25–29). The conclusion of this paper is that *common neurodegenerative diseases of the human CNS and retina including AD, AMD, and PrD, each appear to utilize an overexpressed miRNA-146a in their disease mechanism, and that this anomaly commonly disrupts homeostatic innate-immune signaling to promote an inflammatory phenotype*.

While pro-inflammatory miRNAs such as miRNA-146a are generally considered to be important epigenetic, post-transcriptional regulators of gene expression in both health and disease, it is not often appreciated that these sncRNAs: (i) are very highly selected in their ribonucleotide sequence in mouse and human and exhibit remarkable brain cell and CNS tissue specificity; (ii) are the smallest yet identified ribonucleic acid carriers of highly specific, genetic regulatory information in the human brain and CNS; (iii) are the most abundant extracellular, highly soluble nucleic acids contained in human circulatory fluids including the extracellular fluid (ECF), the cerebrospinal fluid (CSF), and blood serum; and (iv) as such may be capable of spreading genetic signaling information, both homeostatic and pathogenic, among neighboring CNS cells and tissues (20, 44–47). Anti-miRNA-146a (AM-146a) strategies aimed at quenching pathogenic miRNA effects have worked surprisingly well in cell cultures *in vitro*, but their efficacy in progressive neurological disease awaits additional animal experimentation and human clinical trials. Investigations are further warranted for the potential utility of circulating miRNA-146a and its related family members as potential diagnostic biomarkers for AD, AMD, PrD, and perhaps other age-related human neurological diseases with an innate-immune and pro-inflammatory component (31, 48–50).

## Conflict of Interest Statement

The authors declare that the research was conducted in the absence of any commercial or financial relationships that could be construed as a potential conflict of interest.
